# Bi-stream CNN Down Syndrome screening model based on genotyping array

**DOI:** 10.1186/s12920-018-0416-0

**Published:** 2018-11-20

**Authors:** Bing Feng, William Hoskins, Yan Zhang, Zibo Meng, David C. Samuels, Jiandong Wang, Ruofan Xia, Chao Liu, Jijun Tang, Yan Guo

**Affiliations:** 1 College of Education, Zhejiang University, Hangzhou, Zhejiang, 310058 People’s Republic of China; 2 Department of Computer Science and Engineering,University of South Carolina, Columbia, 29208 SC USA; 3 School of Medicine,The University of New Mexico, Albuquerque, 87131 NM USA; 4 Vanderbilt University School of Medicine,Vanderbilt University, Nashville, 37232 TN USA; 5 School of Computer Science and Technology, Tianjin University, 300072, Tianjin, 300072 People’s Republic of China

**Keywords:** Deep learning, Convolutional neural networks, Human down syndrome, Genotyping

## Abstract

**Background:**

Human Down syndrome (DS) is usually caused by genomic micro-duplications and dosage imbalances of human chromosome 21. It is associated with many genomic and phenotype abnormalities. Even though human DS occurs about 1 per 1,000 births worldwide, which is a very high rate, researchers haven’t found any effective method to cure DS. Currently, the most efficient ways of human DS prevention are screening and early detection.

**Methods:**

In this study, we used deep learning techniques and analyzed a set of Illumina genotyping array data. We built a bi-stream convolutional neural networks model to screen/predict the occurrence of DS. Firstly, we built image input data by converting the intensities of each SNP site into chromosome SNP maps. Next, we proposed a bi-stream convolutional neural network (CNN) architecture with nine layers and two branch models. We further merged two CNN branch models into one model in the fourth convolutional layer, and output the prediction in the last layer.

**Results:**

Our bi-stream CNN model achieved 99.3% average accuracies, and very low false-positive and false-negative rates, which was necessary for further applications in disease prediction and medical practice. We further visualized the feature maps and learned filters from intermediate convolutional layers, which showed the genomic patterns and correlated SNPs variations in human DS genomes. We also compared our methods with other CNN and traditional machine learning models. We further analyzed and discussed the characteristics and strengths of our bi-stream CNN model.

**Conclusions:**

Our bi-stream model used two branch CNN models to learn the local genome features and regional patterns among adjacent genes and SNP sites from two chromosomes simultaneously. It achieved the best performance in all evaluating metrics when compared with two single-stream CNN models and three traditional machine-learning algorithms. The visualized feature maps also provided opportunities to study the genomic markers and pathway components associated with Human DS, which provided insights for gene therapy and genomic medicine developments.

## Background

Human Down syndrome (DS) is usually caused by genomic micro-duplications and dosage imbalances of human chromosome 21 [1]. It is associated with many genomic and phenotype abnormalities [2, 3]. Currently, human DS occurs at a very high rate, which is about 1 per 1000 births worldwide [4]. Human DS is also associated with a group of serious diseases, including congenital heart defects, intellectual disability, leukemia, Alzheimer’s disease, Hirschsprung disease, early aging, physical abnormalities, and other abnormalities [1, 5–7]. Current treatments of human DS mainly concentrate on physical therapy [8, 9], emotional and behavioral therapies [10, 11], educational therapy, and early intervention [10, 12]. However, these therapies only have some limited effects that cannot cure DS fundamentally.

DS screening has been studied for more than 50 years. Currently, widely used approaches include combined genomic test [13], blood test [14], sequencing test [15], and ultrasound measurement of nuchal translucency [16]. However, 1/16 of positive screening women may still suffer from further invasive diagnostic procedures, which might result in fetal loss [15, 17]. Therefore, an accurate and error-less DS screening method could significantly reduce the risk of human DS screening procedures.

Recent genome-wide association studies (GWAS) and single nucleotide polymorphisms (SNPs) studies have proved strong correlations between genomic abnormalities and occurrences of different kinds of diseases [18–21]. DS related GWAS studies also showed that SNP variations, gene copy number variations (SNVs), and lots of unidentified genomic variations were associated with the complex genomic disorders and abnormalities of Human DS [22, 23]. However, only a few biomarkers have been discovered to associate with Human DS, such as chorion gonadotropin, unconjugated estriol, and alpha-fetoprotein [24, 25]. Human chromosome 21(Hsa21) encodes more than 500 genes [26, 27] and have various functions, including RNA splicing protein modifiers, cell surface receptors, transcription factors, adhesion molecules, and biochemical pathway components [27, 28]. Currently, 160 of Hsa21 genes have already been annotated as protein-coding genes by SwissProt. Five of them are microRNAs. Most of them have unknown functions [29]. The over-expression of Hsa21 genes results in complex genomic disorders and perturbations of biological processes and pathways [28]. Illumina has introduced a new exome genotyping array technique to identify rare single-nucleotide polymorphisms, which is an alternative technique of high-throughput sequencing. The Vanderbilt University Medical Center and Center for Quantitative Sciences developed an exome chip–processing protocols for this techinique [23].

Machine learning has already been applied to human diseases and genomic pattern predictions [30–32]. Based on our knowledge, only limited types of traditional machine learning techniques have been used in human DS studies [27, 33]. Most of them are performed on mice DS models [23, 27, 34]. Zhao et al. used hierarchical constrained regional model and independent component analysis to detect Human Down syndrome of pediatric patients [35]. Cao et al. used a Naive Bayes model to predict locomotor activities in mice models Ts65Dn and Ts1Cje under the treatments of N-methyl-D-aspartate receptor [34]. Clara et al. designed an unsupervised self-organizing map model to identify biological differences in mice model Ts65Dn [27]. Recently, deep neural networks, especially convolutional and recurrent neural networks, have achieved impressive performances in disease screening, predictions and diagnosis studies [30, 36–38].

In this study, we used convolutional neural networks to construct human Down Syndrome screening/prediction models based on Illumina genotyping array data. Firstly, we built image input data by converting the intensities of SNP sites into chromosome SNP maps. Then we proposed a bi-stream convolutional neural network architecture with nine layers and two branch CNN models, which took two input chromosome SNP maps simultaneously. We also constructed another two single-stream CNN models, which took one chromosome SNP map as input image using the same dataset. Next, we used three traditional machine learning algorithms Random Forest, SVM, and Decision Trees to construct DS screening/prediction models with the same dataset. We evaluated, compared, and analyzed the performance metrics for all models mentioned above. We concluded that our bi-stream CNN model had best performances in all evaluation metrics when compared with other models. At last, we visualized feature maps and learned filters from intermediate layers to study the genomic patterns and correlated gene and SNP variations. We also analyzed and discussed the characteristics and strengths of the bi-stream CNN model.

## Result

### Building human chromosome SNP maps

**Fig. 1:**
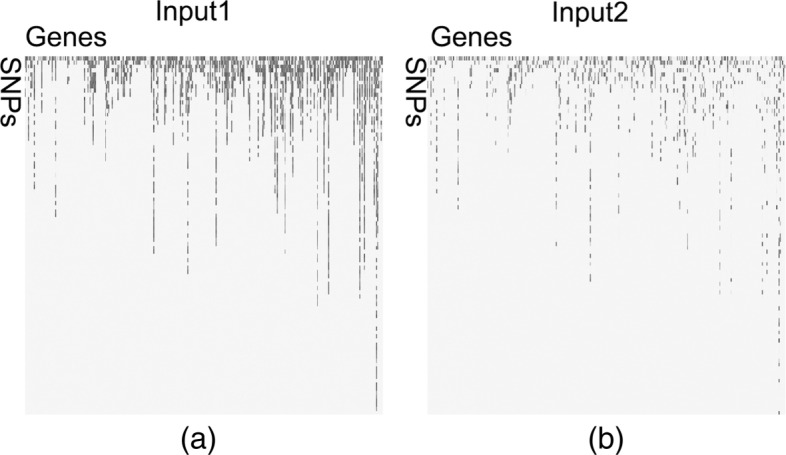
Chromosome SNP maps to represent the intensities of all SNP site on HSA21. Each column represents the information of one single gene located on the chromosome. Each row represents adjacent SNP sites within the same gene. Therefore, each pixel of of the chromosome SNP map is used to represent the intensity of each SNP site of genes

The genotyping dataset used in this study was Illumina exome genotyping array data, which targeted rare single-nucleotide polymorphisms. The dataset contained 378 samples, including 63 DS samples and 315 control samples. Each sample contained the intensity information of 5458 SNPs sites from 321 Hsa21 coding genes. The SNP intensities were normalized to the interval [0,1]. As shown in Fig. 1, we built two chromosome SNP maps to represent the intensities of all SNP site for two Hsa21 chromosomes. Each column of chromosome SNP map represented one single gene. Each row represented adjacent SNP sites within the same gene. Therefore, each pixel could be used to represent the intensity of each SNP site. In this study, we used chromosome SNP maps as input images to construct and evaluated CNN models. For traditional machine learning algorithms, we used original Illumina genotyping array dataset to construct and evaluate the models. For each model construction and evaluation, we did ten parallel experiments with ten sample datasets by randomly sampling the original dataset ten times. Each sample dataset randomly selected 75% data for training and the rest 25% for testing. We calculated average evaluating metrics to provide reliable evaluations.

### Bi-stream convolutional neural network architecture

**Fig. 2:**
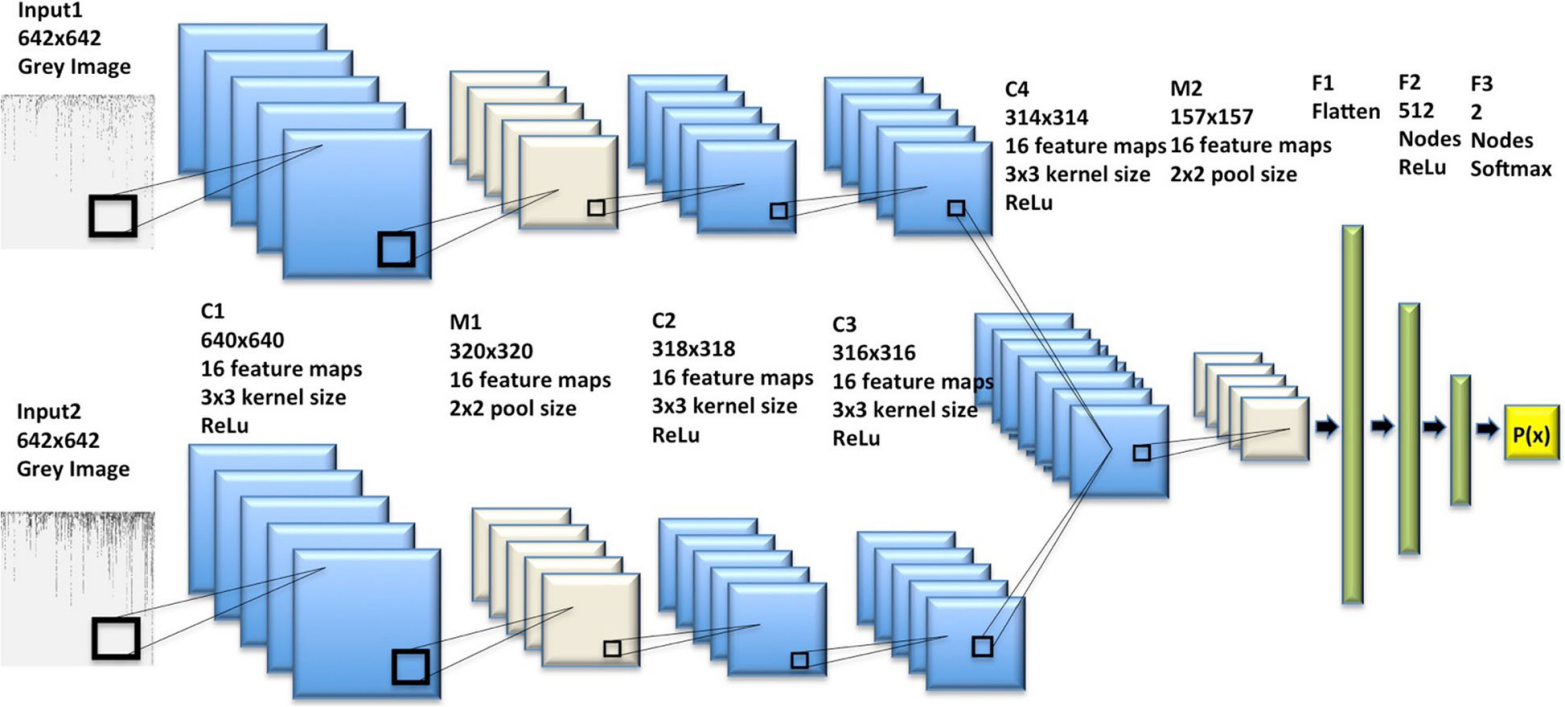
Bi-stream CNN architecture taking two chromosome SNP maps as inputs The upper CNN branch model and the lower CNN branch model both take one chromosome SNP map as input image. We merged two branch CNN models into one CNN model in the fourth convolutional layer C4, which was also followed by a max-pooling layer. Detailed CNN architecture construction and configurations are available in the Method section

Figure 2 showed the architecture of the bi-stream CNN model used in this study, which was merged from two branch CNN models. Each branch model had one input layer, three convolutional layers, and one max-pooling layer. Therefore, our bi-stream CNN model could take two chromosome SNP maps as input images simultaneously. We merged two branch CNN models into one CNN model in the fourth convolutional layer, which was followed by a max-pooling layer. Next, we had another three fully connected layers and one output layer. We added dropouts for each hidden layer for reducing over-fitting. Detailed CNN architecture and configurations were available in the Method section.

### Bi-stream CNN DS screening/prediction model

We first constructed human DS screening/prediction model using bi-stream CNN architecture proposed in the last section. To provide reliable and confidence evaluation, we ran ten parallel experiments on ten randomly sampled dataset and calculated average performance metrics. As shown in Table 1, our bi-stream CNN model achieved 99.3% average accuracy in ten parallel experiments. The average precision, recall, and F-score were 99.2, 98.4, and 99.3%. It was worth to notice that the bi-stream CNN model had very low false-positive and false-negative rates, which were 0.6 and 1.1%. We only mis-predicted five non-DS samples and two DS samples in all ten experiments. Our results showed that the bi-stream CNN architecture could construct very accurate and robust human DS screening/prediction models.

**Table 1:** Evaluation metrics of bi-stream CNN and conventional machine learning models

Models	Evaluation metrics of different models					
	Accuracy	Precision	Recall	F-score	False-positive rate	False-negative rate
Decision tree	96.9(+-1.0)%	94.1%	95.4%	94.6%	2.2%	8.0%
Random forest	97.1(+-0.7)%	94.4%	94.9%	94.7%	1.9%	8.1%
SVM	96.7(+-0.9)%	92.7%	95.9%	94.2%	2.9%	5.3%
Bi-Stream CNN	99.3(+-0.4)%	99.2%	98.4%	99.3%	0.6%	1.1%

### Comparing with traditional machine learning DS screening/screening models

We further applied three different traditional supervised learning algorithms to construct human DS prediction models using the original Illumina genotyping array data with total 5458 SNP features. We also ran ten parallel experiments and further compared the performances with our bi-stream CNN model. Table 1 showed that Random Forest, SVM, and Decision Tree models could achieve very high average accuracies, which were all above 96%. The model built from Random Forest achieved the best performance in all evaluation metrics among all three traditional learning algorithms. Nevertheless, Table 1 also showed that the bi-stream CNN model produced higher accuracy, precisions, recalls, and F-scores when compared with traditional machine learning algorithms. Furthermore, the false negative rates of Random Forest, SVM, and Decision Tree models were very high, which were 8.1, 5.3, and 8.0% respectively. Models with such high false-negative rate were impractical to be applied in real-life clinical prediction and medical practice. However, the bi-stream CNN models achieved significantly better performances in false-positive and false-negative rates, which were only 0.6 and 1.1%. The result above demonstrated that the bi-stream CNN model achieved better performances when compared with the traditional machine learning algorithms. It was more suitable for human DS screening.

### Comparing with single-stream CNN model

Here we built two new single-stream CNN models using the same configurations and datasets with our bi-stream CNN model proposed above. The only difference between bi-stream and single-stream CNN models was that single stream model only had one CNN branch and took one chromosome SNP map as the input image. We further compared and evaluated the performances of two single-stream CNN models. As Table 2 shown, our bi-stream CNN model achieved the best performance over all three models in all evolutionary metrics. The other two single-stream CNN model also achieved over 96% accuracies. However, the recall of the first single-stream model and the precision of the second single-stream model were very low, which were 84.0% and 88.7% respectively. Furthermore, the false positive and false negative rate of the single-stream CNN models were significantly higher than the bi-stream CNN model. In general, our bi-stream CNN model had significantly better performances than the single-stream CNN models. The single-stream models could only extract the genome features from one single chromosome, which completely neglected the genomic patterns from the other one. Therefore, they were not as accurate as the bi-stream CNN model. The bi-stream CNN model was more comprehensive, accurate, and reliable when compared with the single-stream DS prediction models.

**Table 2:** Evaluation metrics of different CNN models

Models	Evaluation metrics of different CNN models					
	Accuracy	Precision	Recall	F-score	False-positive rate	False-negative rate
Bi-Stream CNN	99.3(+-0.4)%	99.2%	98.4%	99.3%	0.6%	1.1%
Single-stream CNN (ChrA)	96.4(+-0.5)%	94.7%	84.0%	96.4%	5.2%	3.2%
Single-stream CNN (ChrB)	96.6(+-0.6)%	88.7%	92.9%	96.6%	11.2%	4.3%

### Visualization of feature maps and trained filters of bi-stream model

**Fig. 3:**
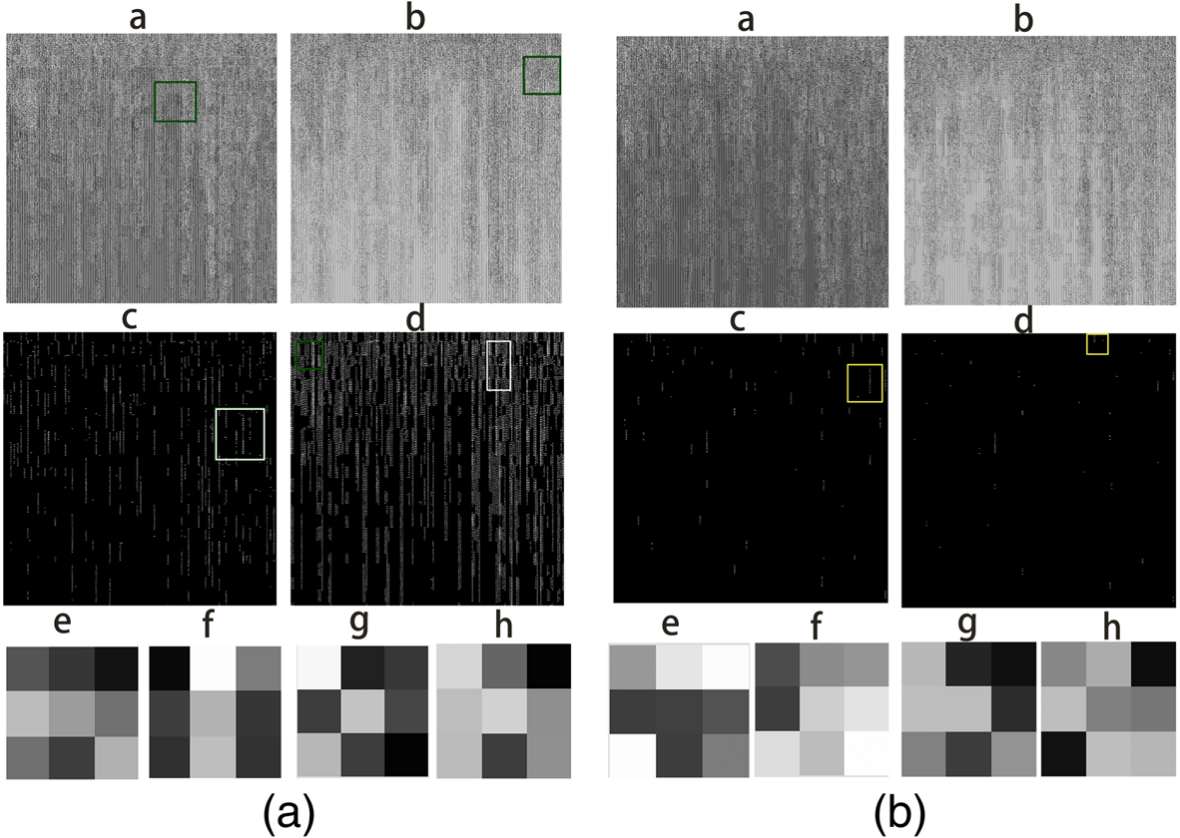
Visualization of feature maps and trained filter weights from convolutional layer C1(shown in Fig. 2). Figure a, b, c and d in figure (**a**) represent four feature maps from convolutional layer C1 of lower branch CNN model (shown in Fig. 2). Figure e, f, g and h in figure (a) are the corresponding 3x3 filters weights of Figure a, b c and d. Figure a, b, c and d in Figure (**b**) represent four feature maps from convolutional layer C1 of the upper branch CNN model. Figure e, f, g and h in figure (**b**) are the corresponding 3x3 filters weights for Figure a, b, c and d

In this section, we visualized the trained filters and feature maps from intermediate convolutional hidden layers of our trained bi-stream CNN model. The bi-stream CNN model had a few advantages when compared with traditional machine learning algorithms. First of all, we used chromosome maps to represent the genotyping array information, which converted one-dimensional genome data to images. Secondly, We used 16 convolutional 3x3 size kernels to capture local genomic features and detect patterns from adjacent genes and SNP sites from two chromosome SNP maps. Thirdly, two branch CNN model could capture the genomic features from two chromosomes at the same time. Figure 3a and b showed the output feature maps and their corresponding trained filters from convolutional layer C1 of each branch CNN models. Some trained filters could highlight the most important and informative SNP sites from the chromosome SNP maps, and neglect less informative ones (marked as yellow squares). The green rectangles showed that our trained filters could sharpen input images and capture local motifs, which represented the correlated variations patterns in genome regions. The bi-stream model could also detect continuous gene and SNP intensity variations by capturing adjacent variation patterns in line(marked as white rectangles). Our bi-stream CNN model could detect the simultaneous or causal SNPs variations in human genomes. These genome characterizations and extracted genomic patterns provided signals to classify DS and normal samples. However, traditional machine learning algorithms tended to build models with a global view from all available features and treated each feature independently. Therefore they were hard to extract signals from regional genomic patterns and correlations between adjacent genes and SNPs sites.

## Discussion

Previous studies illustrated that gene expressions and SNP variations were highly correlated within local genome regions [39–41]. Genome-wide association studies also demonstrated that human DS was usually associated with many gene copy number and SNPs variations, and many unidentified genomic abnormalities [23, 42, 43]. In this study, our bi-stream CNN model could learn the genomic features and associated variations among adjacent genes and SNP sites from chromosome SNP maps. Currently, human DS treatments only have limited effects and can not cure DS fundamentally. There isn’t any clear effect or benefit on human DS treatments using traditional drugs either [44–47]. The feature maps and extracted genome features could identify DS related markers and pathway components. These genome features explained thegenomic characteristics and pathological mechanisms of human DS, which could be further be applied in gene therapy and genetic medicine developments.

An accurate non-invasive DS screening method offers a low-risk way to screen human DS. It helps low-risk patients avoid taking further invasive diagnostic procedures, which might result in fetal loss. Nowadays, genotyping array analyses on fetal genomes could be performed on the trophoblast cells with non-invasive procedures after the fifth week gestation [42, 43]. In this study, we developed a novel method to construct accurate DS screening model by using bi-stream CNN and genotyping array data. The results showed that our bi-stream CNN model had the best performance in every evaluation metric when compared with two single-stream CNN models and three traditional machine learning models. The CNN model achieved over 99.3% accuracies, as well as very low false positive and false negative rates. It was very important to disease prediction and medical practice. Even though traditional machine learning algorithms obtained over 96% accuracies, their high false-negative rates are not suitable for clinical screening tests. Traditional machine learning algorithms treated each SNP sites as single feature independently. They were hard to extract signals from regional genomic patterns and variation correlations between adjacent genes and SNPs sites. Although the single-stream models could extract features and patterns from local genome features and adjacent SNP sites, they could only learn these features from one single chromosome, which completely neglected the genomic patterns of the other one. In deep learning studies, large datasets were great obstacles in the model construction and optimization. We used each pixel to represent the intensities of SNP site, and used chromosome SNP maps to represent the genome information, which significantly reduced data and model complexity. Furthermore, our bi-stream CNN architecture could learn local genomic patterns and extracted regional features, which could also be applied to building prediction models from genotyping array data for more diseases.

## Method

### Data

In this study, the rare single-nucleotide polymorphisms were measured by newly introduced Illumina exome genotyping array technique. Illumina exome genotyping array could identify rare single-nucleotide polymorphisms, which was an alternative technique of high-throughput sequencing. The Vanderbilt University Medical Center and Center for Quantitative Sciences had developed an exome chip–processing protocols for this techinique [23], and provided us the experiment data. The dataset contained the intensity information of total 5458 SNP sites from 321 coding genes on Hsa21 [48]. There were total of 378 samples, including 63 DS samples and 315 control samples.

### Bi-stream CNN architecture

**Fig. 4:**
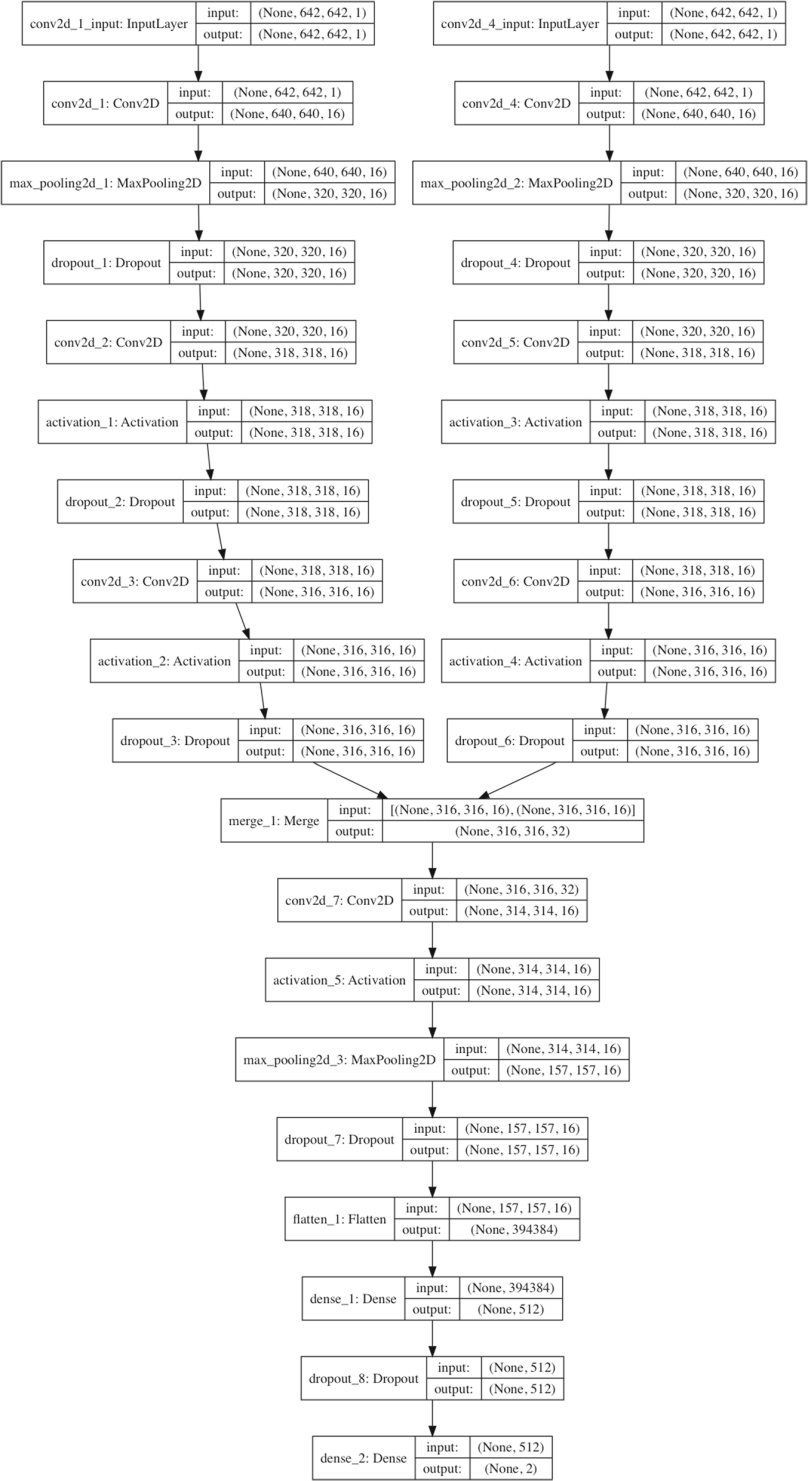
Detailed configurations and structures for each layer of the bi-stream CNN DS prediction/screening model

Our bi-stream CNN model was merged from two branch CNN models. Each branch CNN model had one input layer, three convolutional hidden layers, and one max pooling layer. We fed two input chromosome SNP maps to the two branch CNN models at the same time. Two branch CNN models were further merged into one CNN model in the sixth layer, which was also a convolutional hidden layer. Figure 4 showed the detailed deep neural network structure and configurations for each layer. Detailed information and configurations were shown as below:

Each branch model contained five layers: Layer 1, the input layer took one size 642 ×642 grey chromosome SNP map image as input. Layer 2, one convolutional layer with 16 3*3 filters and ReLu activation. Layer 3, one max pooling layer with 2*2 pool size to down-sample the data, followed by a dropout (0.25) to reduce over-fitting. Layer 4, one convolutional layer with 16 3*3 filters and ReLu activations, followed by dropout (0.25). Layer 5, one convolutional layer with 16 3*3 filters and ReLu activations, followed by dropout (0.25). Next, in layer 6, we merged two branch CNN models into one convolutional layer with 16 3*3 filters and ReLU activations. Layer 7 was another max pooling layer with 2*2 pool scale, followed by dropout (0.25). Layer 8 was a fully connected layer to flatten all features into one-dimension. Layer 9 was a fully connected layer with 512 nodes and ReLU activation. Layer 10 was the output layer with two nodes and Softmax activation. We used stochastic gradient descent optimizer (SGD) and binary cross-entropy as loss function, with a learning rate of 0.01, 1e-6 decay and 0.9 nesterov momentum. We used Tensor-flow and Keras construct all CNN models that used in this study. We use a NVIDIA GeForce GTX TITAN X GPU to build our model on a Ubuntu 14.04.5 LTS machine[48].

### Conventional machine-learning algorithms

In this study, we used Python and Scikit Learn package [49] to implement and construct models for traditional machine learning algorithms SVM, Random Forest, and Decision Tree[49]. SVM was implemented using C-Support Vector Classification algorithm, which used “one-vs-one” scheme. Random Forest and Decision Tree used entropy and Gini impurity to measure features’ splitting qualities. There was no maximum depth limit for Random Forest sub-trees, unless there were less than two samples or all leaves were pruned. We used Classification and Regression Trees algorithm to implement the Decision Tree models. We constructed binary tree with the largest information gains on each splitting node, which was very similar to C4.5 decision tree algorithm. No depth limit was preset before training decision tree models. The maximum features used in model building was set to the total number of features [48].

## Conclusion

In this study, we proposed bi-stream convolutional neural network architecture to construct accurate and robust human Down Syndrome screening and prediction model using Illumina genotyping array data. Our bi-stream CNN model was merged from two branch CNN models, which used two chromosome SNP maps as input images simultaneously. Two branch CNN models were further merged into one CNN model in a deeper convolutional layer. The comparison results showed that the bi-stream CNN model achieved the best performances in all evaluation metrics when compared with other three traditional machine learning algorithms and two single-stream CNN models. The CNN model could achieve 99.3% accuracies with very low false-positive and false-negative rates. Even though the conventional learning algorithms also obtained over 96% accuracies, their high false negative-rates made them hard to be applied in real life clinical screening test. Our bi-stream model used two branch CNN models to learn the local genomic pattern and regional correlations of the adjacent genes and SNPs from two chromosomes simultaneously. However, the single-stream CNN models only learn genomic features from one single chromosome, which completely neglected the genomic patterns of the other chromosome. The genomic patterns, correlated genes and SNPs variation identified by our CNN model provided opportunities to study the genomic markers and pathway components associated with human DS, which could be further applied in gene therapy and genomic medicine developments. Therefore, our method could learn local genomic patterns and extracted regional features from chromosome SNP maps, which could be applied to building prediction models from genotyping array data for more diseases.
